# Supplementary material for: The acute effects of cannabidiol on emotional processing and anxiety: a neurocognitive imaging study
Source: Psychopharmacology (Berl). 2022 Apr 21;239(5):1539–49. doi: 10.1007/s00213-022-06070-3 (PMC9110481; doi:10.1007/s00213-022-06070-3)
Supplement: Supplementary file 1 — Supplementary file1 (DOCX 30 KB) [file 213_2022_6070_MOESM1_ESM.docx]

**SUPPLEMENTARY MATERIALS**

**The acute effects of cannabidiol on emotional processing and anxiety: a neurocognitive imaging study**

**Michael AP Bloomfield^1,2,3,4,5,6^*^#^, Yumeya Yamamori** ^1,7^***, Chandni Hindocha^1,2,4^, Augustus PM Jones^1,8^, Jocelyn LL Yim^1,7,9^, Hannah R Walker^1^, Ben Statton^10^, Matthew B Wall^11^, Rachel H Lees^12^, Oliver D Howes^3,13^, H Valerie Curran^2,4^, Jonathan P Roiser^7^, Tom P Freeman^1,2,12,14^**

1 Translational Psychiatry Research Group, Research Department of Mental Health Neuroscience, Division of Psychiatry, University College London, London, United Kingdom

2 Clinical Psychopharmacology Unit, Research Department of Clinical, Educational and Health Psychology, University College London, London, United Kingdom

3 Psychiatric Imaging Group, Medical Research Council London Institute of Medical Sciences, Imperial College London, Hammersmith Hospital, London, United Kingdom

4 NIHR University College Hospitals London Biomedical Research Centre, University College London, London, United Kingdom

5 The Traumatic Stress Clinic, St Pancras Hospital, Camden and Islington NHS Foundation Trust, London, United Kingdom

6 National Hospital for Neurology and Neurosurgery, University College London Hospitals NHS Foundation Trust, United Kingdom

7 Institute of Cognitive Neuroscience, University College London, United Kingdom

8 Medical Sciences Division, University of Oxford, United Kingdom

9 King's Clinical Trials Unit, King’s College London, London, United Kingdom

10 Medical Research Council London Institute of Medical Sciences, Imperial College London, Hammersmith Hospital, United Kingdom

11 Invicro, Hammersmith Hospital, London, United Kingdom

12 Addiction and Mental Health Group (AIM), Department of Psychology, University of Bath, Bath, United Kingdom

13 Psychosis Studies, King's College London, London, United Kingdom

14 National Addiction Centre, King’s College London, London, United Kingdom

* These authors contributed jointly to the research (for joint first authorship)

*Full inclusion and exclusion criteria*

Inclusion criteria were: i) healthy volunteer; ii) English-speaking; iii) age 18-70 years; iv) right-handed. Exclusion criteria were: i) current use of psychotropic drugs; ii) current/past use of cannabis or cannabidiol; iii) no more than 5 occurrences of recreational drug use other than cannabis; iv) current or history of mood, psychotic, anxiety or substance abuse disorder assessed with an adapted version of the structured clinical interview for DSM-IV (First, Gibbon, Spitzer, Williams, & Benjamin, 1997) (SCID-IV); v) current nicotine dependence defined by a score greater than 3 on the Fagerström Test for Nicotine Dependence (Heatherton, Kozlowski, Frecker, & Fagerstrom, 1991) (FTND); vi) hazardous/harmful alcohol use defined by a score greater than 7 on the Alcohol Use Disorders Identification Test (Saunders & Babor, 1993) (AUDIT); vii) pregnancy; viii) lack of capacity; ix) needle phobia; x) colour blindness, xi) allergies to/unwillingness to take cannabidiol, microcrystalline cellulose, gelatin or lactose; and xii) contraindications to fMRI.

*MRI acquisition and pre-processing procedures*

*MRI acquisition.* Scanning was performed on a Siemens 3T Prisma MRI scanner. A total of 181 whole-brain volumes of 44 axial slices were collected with a T2*-weighted echo-planar imaging (EPI) sequence (TR = 3000 ms, TE = 30 ms, slice thickness = 3 mm, 2 x 2 mm in-plane resolution, phase encoding direction = anterior → posterior, field of view = 250 mm2, matrix size = 64 x 64, flip angle = 90°). A whole-brain structural volume of 176 axial slices was acquired afer the functional scans with a T1-weighted magnetisation-prepared rapid gradient-echo (MPRAGE) sequence (TI = 900 ms, TR = 2300 ms, TE = 2.28 ms, slice thickness = 1 mm, 1 mm2 in-plane resolution, phase encoding direction = anterior → posterior, field of view = 256 mm2, matrix size = 256 x 256, flip angle = 9°).

*Pre-processing.* The fMRI data were pre-processed using MATLAB (The Mathworks Inc., 2017) and Statistical Parametric Mapping (Friston, Ashburner, Kiebel, Nichols, & Penny, 2007) (SPM12). The first four volumes of each functional scan were discarded to allow for T1 equilibration. The scans were then realigned to the new first image using a least-squares approach and a 6-parameter (rigid-body) affine transformation. The dimensions of the functional image voxels were used as translation thresholds (i.e. 2 mm for x and y translations, 3 mm for z translations), and 2° was used as the rotation threshold. No images exceeded these motion thresholds. The scans were normalised to the Montreal Neurological Institute- (MNI) 152 template, using a 12-parameter affine transformation, and smoothed using a 8 x 8 x 8 mm full-width half-maximum Gaussian kernel.

*Non-drug related statistical results*

*A priori outliers.* All behavioural models were re-tested after the exclusion of a) the participant who had breakfast, and b) the two participants who were outside healthy BMI ranges, to determine whether these a priori outliers affected any drug-related effects (main effects of drug or drug interaction effects). As this did not affect any results, the full sample was retained for all tests. Drug administration order as a between-subjects factor also did not affect the results, so are not discussed further.

Since the drug-related results are reported in the main article, here we only report the effects which did not include a factor of drug (i.e. effects only relating to task factors).

*Face rating task*

*Valence.* With respect to valence judgments, there was a significant main effect of emotion (*F_1.16,26.73_* = 263.17, *p* < .001, *η^2^p* = .92, BF_10_ = 2.170x10^64^). Post-hoc tests revealed a significant difference across all conditions (*p*_Holm_ < .001), where happy faces were rated as more positive than neutral faces, and neutral faces were rated as more positive than angry faces. With respect to RTs, there was a main effect of emotion (*F_2,46_* = 12.55, *p* < .001, *η^2^p* = .35, BF_10_ = 549). Post-hoc tests showed greater RTs for happy compared to neutral faces (*p*_Holm_ = .005), and similarly greater for angry compared to neutral faces (*p*_Holm_ < .001). This effect was likely due to the VAS design of responses, where neutral responses did not require as much movement of a visual pointer and therefore time for each response.

*Arousal.* With respect to arousal judgments, there was a main effect of emotion (*F_2, 46_* = 15.12, *p* < .001, *η^2^p* = .40, BF_10_ = 2.317x10^8^). Post-hoc tests revealed significant differences in ratings between happy and neutral, and angry and neutral conditions (*p*_Holm_ < .001), where happy and angry faces were rated as more arousing compared to neutral faces. With respect to RTs, there was a main effect of emotion (*F_2,46_* = 15.77, *p* < .01, *η^2^p* = .41, BF_10_ = 948). Post-hoc tests showed greater RTs for happy compared to neutral faces (*p*_Holm_ = .005), and similarly greater for angry compared to neutral faces (*p*_Holm_ < .001). which were again likely due to the VAS design of responses.

*Mental arithmetic task*

*VAS ‘anxious’ and ‘stressed’.* With respect to anxiety, there was a significant main effect of time (*F_1.67,38.32_* = 24.36, *p* < .001, *η^2^p* = .51, BF_10_ = 5.061x10^14^), where repeated contrasts showed that anxiety significantly increased from post-control to pre-stress (*p* < .001) and from pre-stress to post-stress (*p* = .043). With respect to stress, there was a significant main effect of time (*F_1.49,34.29_* = 28.82, *p* < .001, *η^2^p* = .56, BF_10_ = 1.804x10^16^) where repeated contrasts showed that stress significantly increased from post-control to pre-stress, and from pre-stress to post-stress (*p* < .001).

*VAS ‘calm’ and ‘relaxed’.* With respect to calmness, there was a main effect of time (*F_1.72,39.59_* = 16.23, *p* < .001, *η^2^p* = .41, BF_10_ = 5.954x10^7^), where repeated contrasts showed that calmness decreased from pre-stress to post-stress (*p* < .001). A similar pattern of results was observed for relaxedness: there was a main effect of time (*F_1.61,37.07_* = 16.40, *p* < .001, *η^2^p* = .42, BF_10_ = 7.756x10^7^), where repeated showed that relaxedness significantly decreased from post-control to pre-stress scores (*p* = .011), and pre-stress to post-stress scores (*p* = .001).

*Physiological measures.* With respect to SBP, there was a significant main effect of time (*F_3,69_* = 2.88, *p* = .042, *η^2^p* = .11, BF_10_ = 0.662), where repeated contrasts showed that SBP significant increased from post-control to pre-stress (*p* = .005). There was no effect of time for DBP (*F_3,69_* = 1.90, *p* = .138, BF_10_ = 0.357). With respect to HR, there was a significant main effect of time (*F_3, 69_* = 8.08, *p* < .001, *η^2^p* = .26, BF_10_ = 186). Inspection of the repeated contrasts showed that HR significantly changed across adjacent measurements: HR increased from pre-control to post-control (*p* = .046), increased from post-control to pre-stress (*p* = .006) and decreased from pre-stress to post-stress (*p* = .004).

*Subjective and physiological measures*

*VAS ‘anxious’ and ‘happy.* A main effect of time was observed (*F_2.91,66.94_* = 3.36, *p* = .025, *η^2^p* = .13, BF_10_ = 1.040), where anxiety significantly decreased from baseline to 1 h post-drug administration (*p* = .018). There was no effect of time for ‘happy’ scores (*F_2.80,64.33_* = 2.28, *p* = .092, BF_10_ = 0.163).

*Physiological measures.* There was no effect of time for SBP (*F_2.72,62.52_* = 2.15, *p* = 108, BF_10_ = 0.391). A significant main effect of time was observed for DBP (*F_4,92_* = 3.99, *p* = .005, *η^2^p* = .15, BF_10_ = 4.814), where DBP changed between each time-point except for between 1 and 2 h post-drug administration (*p* < .05). A main effect of time on HR was also observed (*F*_4,92_ = 14.98, *p* < .001, *η^2^p* = .39, BF_10_ = 6.389x10^8^), where HR changed between each time point except for between 1 and 2 h post-drug administration (*p* < .05).
